# Varied Clinical Features and Outcomes of Autoimmune Encephalitis: A Retrospective Case Series

**DOI:** 10.7759/cureus.76389

**Published:** 2024-12-25

**Authors:** Aruna Kumari Kotteti, Elanthiraiyan Govindaswamy Chelvakumar, Robert Wilson Sundaram, Kalpana Radhakrishnan, Arunan Subbiah

**Affiliations:** 1 Neurology, SRM Medical College Hospital and Research Centre, SRM Institute of Science and Technology, Chengalpattu, IND

**Keywords:** anti-gamma aminobutyric acid receptor antibodies, anti hu(antineuronal nuclear antibody-type 1), anti ma(neuronal antibody or purkinje cell antibody), anti n-methyl-d-aspartate receptor antibodies, autoimmune encephalitis, cognitive decline, contactin-associated protein-like 2, leucine-rich glioma-inactivated 1 (lgi1) antibody, α-amino-3-hydroxy-5-methyl-4-isoxazolepropionic acid receptor

## Abstract

Introduction: This study discusses the various clinical profiles, investigatory findings, treatment responses, and prognosticating factors in seven cases of autoimmune encephalitis (AE).

Methods: The clinical records of seven AE patients admitted to the Neurology Department, SRM Medical College Hospital and Research Centre, Chennai, from July 2022 to December 2023 were retrospectively analyzed.

Results: The patients' ages ranged from 18 to 35, and all experienced seizures. Only two patients exhibited cognitive decline and psychiatric problems. All seven cases were clinically diagnosed as AE: three were positive for anti-N-methyl-D-aspartate receptor (anti-NMDAR) antibodies, while the AE panel was negative in the remaining patients. In all cases, contrast imaging of the brain on MRI appeared normal.

Conclusion: Patients with AE need to be diagnosed early because prompt immunosuppressive therapy effectively reduces morbidity and mortality. A detailed history, clinical examinations, and relevant investigations are required. Mild and atypical forms with negative autoimmune antibody panels with relatively good outcomes are increasingly encountered.

## Introduction

Autoimmune encephalitis (AE) is increasingly recognized as an important non-infectious encephalitic disorder that may be reversible [1]. It typically presents with acute, uncontrolled seizures, memory impairment, and psychiatric symptoms that evolve over a few weeks to months.

Though the exact mechanism of AE is not yet fully understood, it is known that autoimmune antibodies target cell surface or intracellular neuronal proteins, leading to widespread inflammation. AE affects various parts of the nervous system, including the limbic and spinal cord.

There are several subtypes, with the most common being anti-N-methyl-D-aspartate receptor (anti-NMDAR) encephalitis, anti-gamma-aminobutyric acid B receptor (GABA-B-R) encephalitis, anti-leucine-rich glioma-inactivated 1 (LGI1) encephalitis, and anti-contactin-associated protein-like 2 (CASPR2) encephalitis [2,3]. Although AE is becoming more recognized, delayed or missed diagnoses often result in negative outcomes. Recent clinical reviews provide a wide range of non-infectious differential diagnoses for viral encephalitis but frequently overlook established immunological causes, such as anti-NMDAR encephalitis. We present seven cases that illustrate the diversity of presentations, the causes of adverse events, how these events were managed, and the outcomes.

## Materials and methods

Study design and setting

This retrospective study was conducted in the Neurology Department, SRM Medical College Hospital and Research Centre, India. Clinical records of patients diagnosed with AE for a duration of 18 months, from July 2022 to December 2023, were analyzed.

Patient selection

Patients included in the study were diagnosed with AE based on clinical criteria, including symptoms of seizures, cognitive decline, psychiatric symptoms, and/or positive autoimmune antibody results. Inclusion criteria included patients aged 18-35 years admitted within the specified period. Patients with other neurological conditions or incomplete medical records were excluded.

Data collection

Data were collected from electronic medical records, including demographic information, clinical presentations, laboratory findings, neuroimaging results, treatment protocols, patient outcomes, anti-NMDAR antibody results, and MRI findings.

Outcome measures

Primary outcomes analyzed included treatment response, seizure control, cognitive and psychiatric improvements, and overall prognosis. Prognostic factors were identified based on clinical features and treatment responses.

Statistical analysis

Data were analyzed using descriptive statistics to summarize patient demographics, clinical features, and outcomes.

## Results

Case 1

**Figure 1:**
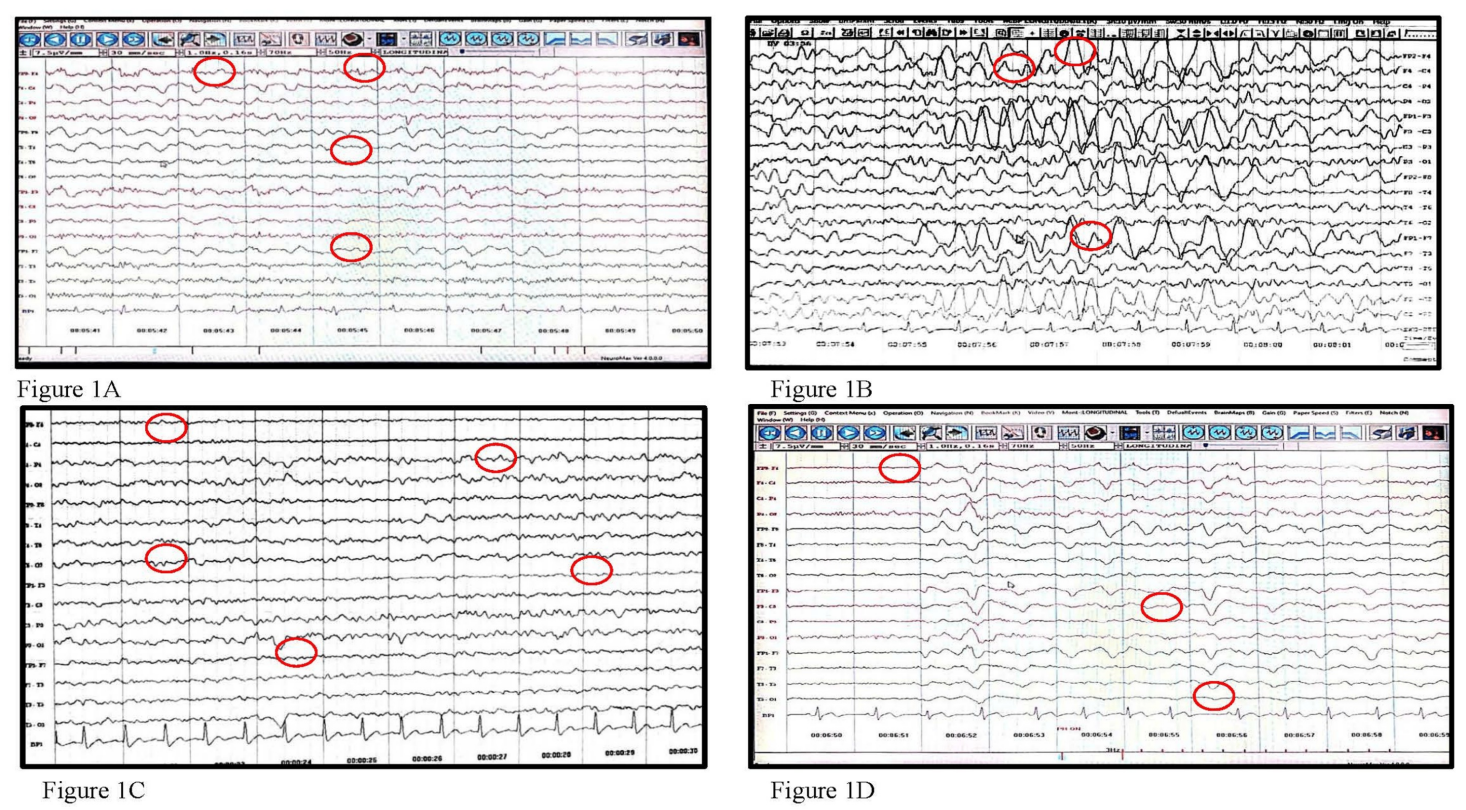
(A) EEG showing bifrontal slowing for Case 1, (B) EEG showing left hemisphere seizure activity for Case 2, (C): EEG showing bifrontal left temporoparietal occipital dysfunction for Case 4, and (D) EEG showing mild cerebral dysfunction for Case 5 EEG: electroencephalogram

A 25-year-old female presented with fever and neck pain for 10 days, bilateral tonic-clonic seizures for three days, and memory disturbances. She had visual hallucinations with generalized choreiform movements. On examination, the patient is conscious, febrile, confused, and neurologically stable. All baseline investigations were normal. CSF analysis was normal, including the CSF viral panel. EEG showed bilateral frontal slowing (Figure 1A). Neuroimaging was normal. The patient was treated with methylprednisolone, acyclovir, and levetiracetam and gradually improved.

Case 2

The 31-year-old male patient was seen with complaints of raised body temperature, altered sensorium with a headache for two days, and two episodes of seizures. All routine investigations were within normal limits. Chest X-ray (CXR) and ultrasonogram (USG) abdomen normal. They were neuroimaging normal. EEG showed left focal seizure activity (Figure 1B). CSF analysis routine glucose 30 mg/dl protein was 60 mg%, cell count was within normal limits, and CSF viral panel reports normal. The patient was treated with dexamethasone and acyclovir.

Case 3

A 22-year-old male patient presented with a fever, altered behavior, vomiting for five days, and multiple episodes of seizures. He exhibited irrelevant speech and was uncooperative during the examination. All routine investigations were within normal limits. CXR, USG abdomen, neuroimaging, and CSF analysis were normal; however, the NMDA receptor antibody test was positive. The patient was treated with steroids, antiepileptics, IVIG at a dose of 0.4 mg/kg/day for five days, and two doses of rituximab. Gradual clinical improvement was observed.

Case 4

A 25-year-old man presented with fever, headache, and two episodes of bilateral tonic-clonic seizures. The patient exhibited cognitive decline, with a MOCA score of 20/30. CSF analysis and neuroimaging results were normal. However, EEG revealed bilateral frontal, left temporal, parietal, and occipital dysfunction (Figure 1C). The patient was treated with steroids, acyclovir, and levetiracetam. Following treatment, the patient's general condition and cognition improved, with a MOCA score of 28/30.

Case 5

An 18-year-old female patient was brought in with a fever lasting 10 days and multiple episodes of seizures over the past two days. Evaluation for paraneoplastic involvement was negative. CSF analysis revealed NMDA receptor antibody positivity. EEG showed mild cerebral dysfunction (Figure 1D). Treatment included corticosteroids, IVIG administered at a dosage of 0.4 mg/kg per day for five days, and levetiracetam. The patient's general condition gradually improved.

Case 6

**Figure 2:**
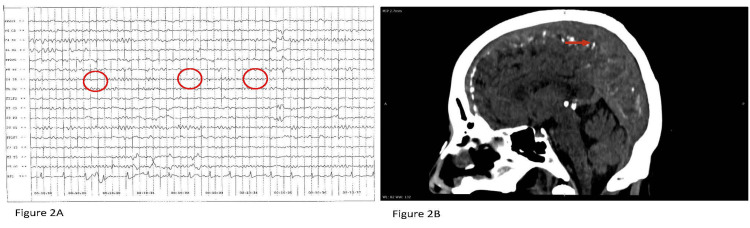
(A) EEG showing occasional right temporal slowing for Case 6 and (B) CT brain showing dural-based calcification for Case 6 EEG: electroencephalogram

A 34-year-old woman presented with complaints of fever, giddiness, headache, and slurred speech. On admission, the patient was afebrile and hemodynamically stable. A nervous system examination revealed a power of 4/5 in the right upper and lower limbs, along with cerebellar signs, including ataxia. CXR and USG abdomen were normal. An EEG showed occasional right temporal slowing (Figure 2A). Routine blood investigations were within normal limits. CSF analysis revealed the following: glucose, 72 mg/dL; protein, 26.53 mg/dL; total cell count, no cells; acellular smear; creatine phosphokinase; ammonia, 190 µmol/L; and a negative viral meningitis panel. A CT scan of the brain showed multiple dural-based calcifications (Figure 2B). Further imaging was normal. The patient was treated with antiviral agents and steroids. Her general condition gradually improved.

Case 7

A 20-year-old woman was admitted to the hospital in a deeply confused state with behavioral changes. The patient was intubated due to a rapid decline in consciousness. All routine examinations were within normal limits. Neuroimaging findings were normal, and EEG results were unremarkable. CSF analysis revealed the presence of anti-NMDA antibodies. The patient’s clinical status significantly improved following treatment with intravenous methylprednisolone, IVIG, and oral corticosteroids. Her general condition gradually improved over time.

Table 1 summarizes the data of all seven patients who presented with clinical features suggestive of AE.

**Table 1:** Demographical, clinical, investigatory findings, and radiological data with treatment outcomes M: male, F: female, EEG: electroencephalogram, MRI: magnetic resonance imaging, CSF: cerebrospinal fluid, NMDA receptor: N-methyl-D- aspartate receptor antibody, MV: mechanical ventilator, IVIG: intravenous immunoglobulin

	Case 1	Case 2	Case 3	Case 4	Case 5	Case 6	Case 7
Age (years)	25	31	22	25	18	34	20
Gender	F	M	M	M	F	F	F
Initial presentation	Epileptic seizure	Epileptic seizure	Epileptic seizure	Psychiatric symptoms	Epileptic seizures	Speech disturbances	Psychiatric symptoms
Seizures	Present	Present	Present	Present	Present	Present	Present
Psychiatric and behavioral abnormalities	-	-	-	Present	-	-	Present
Cognitive impairment	-	-	-	Present	-	-	Present
MRI brain	No abnormality detected	No abnormality detected	No abnormality detected	No abnormality detected	No abnormality detected	No abnormality detected	No abnormality detected
CSF	Within normal limits	Protein elevated	NMDA receptor-positive	Within normal limits	NMDA receptor-positive	Within normal limits	NMDA receptor-positive
EEG	Bilateral frontal slowing	Left focal seizure activity	Generalized slowing	Bilateral frontal and left temporoparietal occipital dysfunction	Mild cerebral dysfunction	Occipital right temporal slowing	Normal
Treatment outcome	Glucocorticoids improved	Glucocorticoids improved	Glucocorticoids and IVIG improved	Glucocorticoids improved	Glucocorticoids and IVIG improved	Glucocorticoids improved	Glucocorticoids and IVIG improved
MV	_	_	_	_	_	_	Patient on MV
Cancer screening	_	_	_	_	_	_	_

## Discussion

Patients presenting with symptoms of encephalitis pose significant diagnostic challenges. The prevalence of viral encephalitis in India, based on the number of patients admitted to hospitals, was approximately five per 100,000 between 1995 and 2015. The incidence rate of AE has increased from five to 10 per 100,000 due to improved recognition of the disorder [4,5]. This condition affects specific demographics, including both young and elderly patients. AE should be considered early in cases of fever with no obvious infectious etiology. Although autoimmune disorders are an uncommon cause of illness, recent advances in diagnostic serology, particularly the detection of antibodies, have made their diagnosis more accessible.

The following cases, spanning 18 months, illustrate various forms of AE. Cases 3, 5, and 7 reported anti-NMDAR encephalitis, with Case 3 exhibiting minor short-term memory impairment. Other cases presented with non-infectious, presumed AE that responded to immune-modulating treatments but lacked identifiable autoimmune antibodies. Important tools for diagnosis include recognizing associated symptoms and clinical signs and conducting relevant investigations. Early identification and intensive treatment- including corticosteroids, plasma exchange, IVIG, immunomodulatory medications, and cyclophosphamide- can effectively manage AE. Prompt intervention may result in the complete reversal of the condition and significantly accelerate patient recovery.

The preliminary assessment of a patient exhibiting encephalitis typically includes a contrast-enhanced brain CT scan, lumbar puncture for cell count, protein and glucose levels, a viral panel, serum glucose correlation, bacterial infection assays, an acid-fast bacilli smear, and mycobacterial culture. A brain MRI should also be obtained, and an EEG may be appropriate in certain circumstances. Some findings from these tests can overlap between infectious and AE cases.

Acute or prodromal syndromes may present with headache, low-grade fever, and nonspecific viral symptoms such as anorexia, vomiting, nausea, and upper respiratory issues. Many patients may present with psychiatric symptoms, leading to their retention in psychiatric care for up to two weeks before a definitive presentation [6]. The initial symptoms of anti-NMDAR encephalitis are often psychiatric, commonly including anxiety, paranoia, sleep disturbances, elaborate delusions, and manic behavior. Additionally, speech and language disorders can occur. The spectrum of echolalia ranges from mutism to memory impairment.

Rapid progression is characterized by reduced responsiveness, autonomic instability, and dyskinesias following the initial phase. This progression may involve both complex and motor seizures, with dyskinesias presenting as evident orolingual-facial movements. Other reported symptoms in AE include stiffness, opisthotonus, limb and trunk choreoathetosis, dystonia, and oculogyric crises [7,8], which can appear simultaneously or alternately during the illness. Approximately 70% of patients experience centrally generated hypoventilation and myoclonus, often necessitating admission to the intensive care unit. Diagnostic serology, particularly the detection of antibodies, is essential.

Recording all relevant symptoms, signs, and examinations is crucial for facilitating an early diagnosis of AE. Anti-NMDAR encephalitis affects men in 31% of cases, although it predominantly affects females with co-occurring ovarian teratomas [9-13]. Children and teenagers account for a significant 42% of patients with anti-NMDAR encephalitis [14,15]. The antigens for NMDA receptor-specific antibodies target the NR1 subunit. The reduction in NMDA receptor surface density and synaptic localization, linked to antibody titers [16-19], results in decreased GABA release from presynaptic neurons, leading to enhanced excitotoxicity, glutamate release, and dopamine dysregulation. A notable difference in presentation between adults and children is that hypoventilation occurs less frequently and is generally milder in children, manifesting as breath-holding episodes or autonomic dysfunction.

Possible features in patients with anti-NMDAR encephalitis include CSF and MRI abnormalities. Abnormal CSF is present in approximately 80% of patients with anti-NMDAR encephalitis, with most experiencing lymphocytic pleocytosis. More than half exhibit elevated levels of certain chemicals in their CSF, such as protein. Antibodies may be detected in serum and CSF, with varying levels. Individuals who respond well to treatment often have lower titers of anti-NMDA antibodies compared to their pre-treatment levels.

Brain MRI may not reveal significant abnormalities, while EEGs commonly show diffuse or frontotemporal slow or chaotic (delta-theta) brain wave patterns without epileptic discharges. Despite normal MRI findings, EEG results may demonstrate excess beta waves along with marked diffuse delta waves. The principal underlying cause of symptoms is widely believed to be anti-NMDAR encephalitis, which often responds well to steroid treatment.

Histological investigations of brain specimens typically do not reveal recognizable changes. It is estimated that approximately 71% of teratomas identified in females with anti-NMDAR encephalitis are benign. In cases where such tumors are detected, a combination of immunotherapy and surgical resection has been associated with better outcomes. Notably, about 42% of patients in other series reported no detectable tumors. Treatment with IVIG and high-dose steroids has been shown to improve outcomes.

However, there is a considerable risk of relapse associated with these treatments. Therefore, tumor resection is indicated if a tumor is detected. Anti-NMDA receptor encephalitis is treated with high-dose steroids [20-22], and IVIG [23-26]. Second-line treatments may include rituximab, cyclophosphamide, or plasma exchange. Third-line agents may involve proteasome inhibitors (bortezomib) [27-28] and IL-6 inhibitors (tocilizumab) [29-30]. Aside from anti-NMDAR, other forms of AE, such as anti-LGI1 or CASPR2, can manifest similarly, but we did not find these conditions in our patients. Disorders such as anti-AMPA, anti-GABA, anti-Hu, and anti-Ma2 receptor antibodies can also present in this manner.

Overall, a synthesis of historical, clinical, and electroencephalographic data is essential. CSF findings can indicate a diagnosis of AE even in the absence of positive auto- or diagnostic serology. The cases presented here, encountered over 18 months, highlight the necessity of treating patients with AE aggressively, promptly, and appropriately to achieve the best outcomes. This study had several limitations: it is a retrospective study with a small sample size, the data are limited to a single hospital, and there was a lack of follow-up for the patients.

## Conclusions

AE can manifest similarly to other forms of encephalitis, including infectious causes. However, NMDA and other AEs may exhibit some unique characteristics. Of the seven cases reviewed, six showed excellent responses to treatment, while one case experienced minor short-term memory impairment. Treatment should begin as early as possible with intravenous steroids, intravenous immunoglobulin, and plasma exchange. Second-line drugs, such as rituximab and cyclophosphamide, may be used if the condition is refractory to first-line agents. Appropriate treatment and timely management of the patient led to improved outcomes, with most cases resulting in complete recovery.

Mild and atypical forms of AE, with negative autoimmune antibody panels due to probable unidentified antibodies, are increasingly recognized. Patients with these milder forms typically recover completely with steroids alone. New antibodies may be identified and added to the list in the future. The importance of this study lies in the early diagnosis and prompt immunosuppressive therapy for AE, which helps reduce morbidity and mortality. Early identification and immediate intervention based on patient history, clinical symptoms, signs, and investigations are crucial for survival and prognosis.
